# Outer membrane permeability of mcr-positive bacteria reveals potent synergy of colistin and macromolecular antibiotics against colistin-resistant *Acinetobacter baumannii*

**DOI:** 10.3389/fmicb.2024.1468682

**Published:** 2024-11-19

**Authors:** Meisong Li, Furong Ma, Hui Zhao, Dianrong Zhou, Lujie Liang, Runling Lv, Jiachen Li, Yaxuan Wang, Lin Xu, Chenfei Liu, Guo-Bao Tian, Siyuan Feng, Yong Xia

**Affiliations:** ^1^Department of Clinical Laboratory Medicine, Guangdong Provincial Key Laboratory of Major Obstetric Diseases, Guangdong Center for Provincial Clinical Research Obstetrics and Gynecology, The Third Affiliated Hospital, Guangzhou Medical University, Guangzhou, China; ^2^School of Pharmacy, Guangzhou Xinhua University, Guangzhou, China; ^3^Department of Immunology and Microbiology, Zhongshan School of Medicine, Sun Yat-sen University, Guangzhou, China; ^4^Advanced Medical Technology Center, The First Affiliated Hospital, Zhongshan School of Medicine, Sun Yat-sen University, Guangzhou, China; ^5^Key Laboratory of Tropical Diseases Control (Sun Yat-sen University), Ministry of Education, Guangzhou, China

**Keywords:** *Acinetobacter baumannii*, colistin, rifampicin, MCR, outer membrane permeability

## Abstract

Colistin (CT) is the last-resort of antibiotic against multidrug-resistance (MDR) *Acinetobacter baumannii* (*A. baumannii*) infection. However, colistin resistance is increasingly reported in *A. baumannii* isolates partially due to the global emergence and dissemination of plasmid-borne mobile colistin resistance (*mcr*) gene and is a threat to human health. Thus, available treatment strategies urgently required in the fight against colistin-resistant *A. baumannii*. Here, we showed that *mcr* confers damaged outer membrane (OM) permeability in *A. baumannii*, which could compromise the viability of *A. baumannii*. Consistently, *A. baumannii* with colistin resistance exhibits increased susceptibility to macromolecular antibiotics such as rifampicin (RIF) and erythromycin (ERY). Moreover, the combination therapy of colistin and rifampicin demonstrates efficacy against colistin-resistant *A. baumannii*, regardless of the presence of *mcr*. Altogether, our data suggest that the synergy of colistin in combination with macromolecular hydrophobic antibiotics poses a promising therapeutic alternative for colistin-resistant *A. baumannii*.

## 1 Introduction

*Acinetobacter baumannii* is identified as a clinically significant opportunistic pathogen causing extensively nosocomial infections, especially in intensive care unit (ICU) patients, patients with prolonged hospitalization and patients undergoing central vascular catheterization and tracheostomy (Morris et al., 2019; Ayoub Moubareck and Hammoudi Halat, 2020). *A. baumannii* is intrinsically resistant to penicillins and cephalosporins, which were previously considered the first-line treatments for *A. baumannii* infections (Dijkshoorn et al., 2007). Except that, *A. baumannii* are resistant to almost all available antibiotics (Gordon and Wareham, 2010). Hence, colistin (also known as polymyxin E), which target the negatively charged phosphate groups of lipid A, is considered one of the final therapeutic options for managing MDR *A. baumannii* infections (Novović and Jovčić, 2023).

The recent surge in colistin usage in clinical practice has led to the rapid spread of resistance in *A. baumannii* (Novović and Jovčić, 2023). There are two primary mechanisms of colistin resistance in *A. baumannii*: complete loss or modifications of the target Lipopolysaccharide (LPS), resulting in the elimination or reduction of its negative charge (Olaitan et al., 2014). Among these, plasmid-mediated colistin resistance encoded by *mcr* genes has been identified as a significant factor driving rapid dissemination through horizontal gene transfer in *A. baumannii*. Since the first mobilized colistin resistance gene, *mcr-1*, was founded in *Escherichia coli* (*E. coli*) and *Klebsiella pneumoniae* (*K. pneumoniae*) in China in 2016 (Liu et al., 2016), *mcr*-positive *A. baumannii* has been isolated from various sources: human feces, human clinical samples (Martins-Sorenson et al., 2020), livestock, food origin, aquaculture products, retail meat etc. (Hameed et al., 2019; Kareem, 2020). The *mcr-1* and *mcr-4.3* are most commonly detected in *A. baumannii* (Novović and Jovčić, 2023). The rapid spread of colistin resistance in *A. baumannii* has made it essential to thoroughly investigate new potential last-resort therapeutic options.

Recently, promising results have been demonstrated *in vitro* for cefiderocol (Stefano Stracquadanio et al., 2022), intravenous fosfomycin (Marino et al., 2022), and combination therapy with sulbactam–durlobactam (Karlowsky et al., 2022) in the treatment of infections caused by colistin-resistance *A. baumannii*. Macromolecular-hydrophobic antibiotics such as rifampicin and erythromycin have shown significant synergy with colistin against *mcr-1*-positive *Enterobacteriaceae*, like *E. coli* and *K. pneumoniae* (MacNair et al., 2018). MacNair et al. propose that the mechanism behind this antibiotic potentiation is due to MCR-1 providing minimal protection against outer membrane perturbation. Indeed, concurrently conferring colistin resistance to the bacteria, MCR-1 disrupts lipid homeostasis, affecting outer membrane permeability, and subsequently leading to compromised viability of *E. coli* and *K. pneumoniae* (Feng et al., 2022). Similar to the impact of MCR, colistin resistance mediated by mutation of *lpxA* results in the complete loss of LPS/Lipooligosaccharide (LOS), leading to a reduction in membrane integrity of *A. baumannii* (Moffatt et al., 2010). However, it remains unclear whether MCR influences the viability of *A. baumannii* and subsequently affects the effectiveness of synergy antibiotic treatment.

Here, we show that the increased OM permeability affected the viability of *mcr-1.1* and *mcr-4.3* positive *A. baumannii* strains in the stationary phase. Moreover, the damage to the OM leads to an increased sensitivity of *mcr*-positive *A. baumannii* to a variety of macromolecular antibiotics such as rifampicin and erythromycin. Consistently, colistin potentiates rifampicin and erythromycin in colistin-resistance *A. baumannii* with or without *mcr* gene. Our study would help to develop therapeutic strategies to improve effectiveness of treatment for *A. baumannii* infections.

## 2 Materials and methods

### 2.1 Bacterial strains and plasmids

The bacterial strains and plasmids used in this study are listed in Supplementary Table S1. The primers used in this study are listed in Supplementary Table S2. Unless otherwise stated, all *A. baumannii* strains were grown in Luria–Bertani (LB) broth or on LB agar plates at 37°C. Antibiotics and other chemicals were used at the following final concentrations: gentamicin, 10 μg/mL; colistin, 4 μg/mL; kanamycin, 50 μg/mL.

### 2.2 Strain construction

To generate pMMB67EH-NP-*mcr-1.1* and pMMB67EH-NP-*mcr-4.3*, the *mcr-1.1/mcr-4.3* gene and their native promoter were amplified from an *mcr*-1-carrying IncX4 (Feng et al., 2018) plasmid and *mcr-4.3* carrying plasmid (Ma et al., 2019) and then cloned into pMMB67EH-spy, a low-copy number plasmid (containing the RSF1010 origin of replication). The map of plasmids and recombinant plasmids used for constructing *mcr*-positive *A. baumannii* strains are listed into Supplementary Figure S1.

### 2.3 Growth kinetics

The *A. baumannii* strains were grown to mid-log phase (OD_600_ = 0.6–0.8) in LB medium. The cultures were pelleted, washed once with PBS, adjusted to an OD_600_ of 0.5 with PBS, and diluted 1: 10. Then, 10 μL of the diluted culture was added into 90ul of LB. The OD_600_ of the culture within 24 h was determined using a microplate reader (BioTek). Three replicates were analyzed for each strain.

### 2.4 Antimicrobial susceptibility testing

The MIC of the antimicrobial agents against *A. baumannii* with or without MCR were determined using the standard broth microdilution method, according to the CLSI 2020 guidelines. In brief, all antibiotics were 2-fold diluted in Mueller-Hinton broth (MHB), and 90 μL of this solution was mixed with 10 μL of diluted culture, containing approximately 1.5 × 10^6^ CFU/mL in a 96-well microtiter plate. After 16–20 h of incubation at 37°C, the MIC values were defined as the lowest concentration of antibiotics with no visible bacterial growth. Experiments were performed with 3 biological replicates.

### 2.5 *In vitro* competition assays

*In vitro* competition experiments were used to measure the competitiveness of the ATCC 17978 (*mcr-1.1*/pMMB67EH), ATCC 17978 (*mcr-4.3*/pMMB67EH), and ATCC 17978 (pMMB67EH only). The *mcr*-positive strains (both resistant to gentamicin and colistin) were competed against ATCC 17978 (pMMB67EH only, only resistant to gentamicin) and plating assays on LB agar plates with colistin/gentamicin or gentamicin was used to measure changes in the CFU of the two strains during competition. All competitions were carried out in M9 medium with three replicates per strain. The bacteria were cultured overnight in LB supplemented with the appropriate antibiotics. After the cell density was normalized (OD_600_ = 0.5), the bacteria solution was diluted 1: 100 in M9 broth and mixed at 1: 1 ratio. Cultures were incubated at 37°C with shaking (220 rpm). After 12, 24, 48, 72 h, aliquots were serially diluted 10-fold in PBS, and 10 μL of each dilution was spotted onto LB agar with colistin/gentamicin (CFU *_mcr_*) or gentamicin (CFU _total_). The plates were incubated at 37°C and enumerate the rate by CFU count after 16 h: rate (%) = CFU *_mcr_*/CFU _total_ * 100%.

### 2.6 SDS sensitivity assay

**Figure 1:**
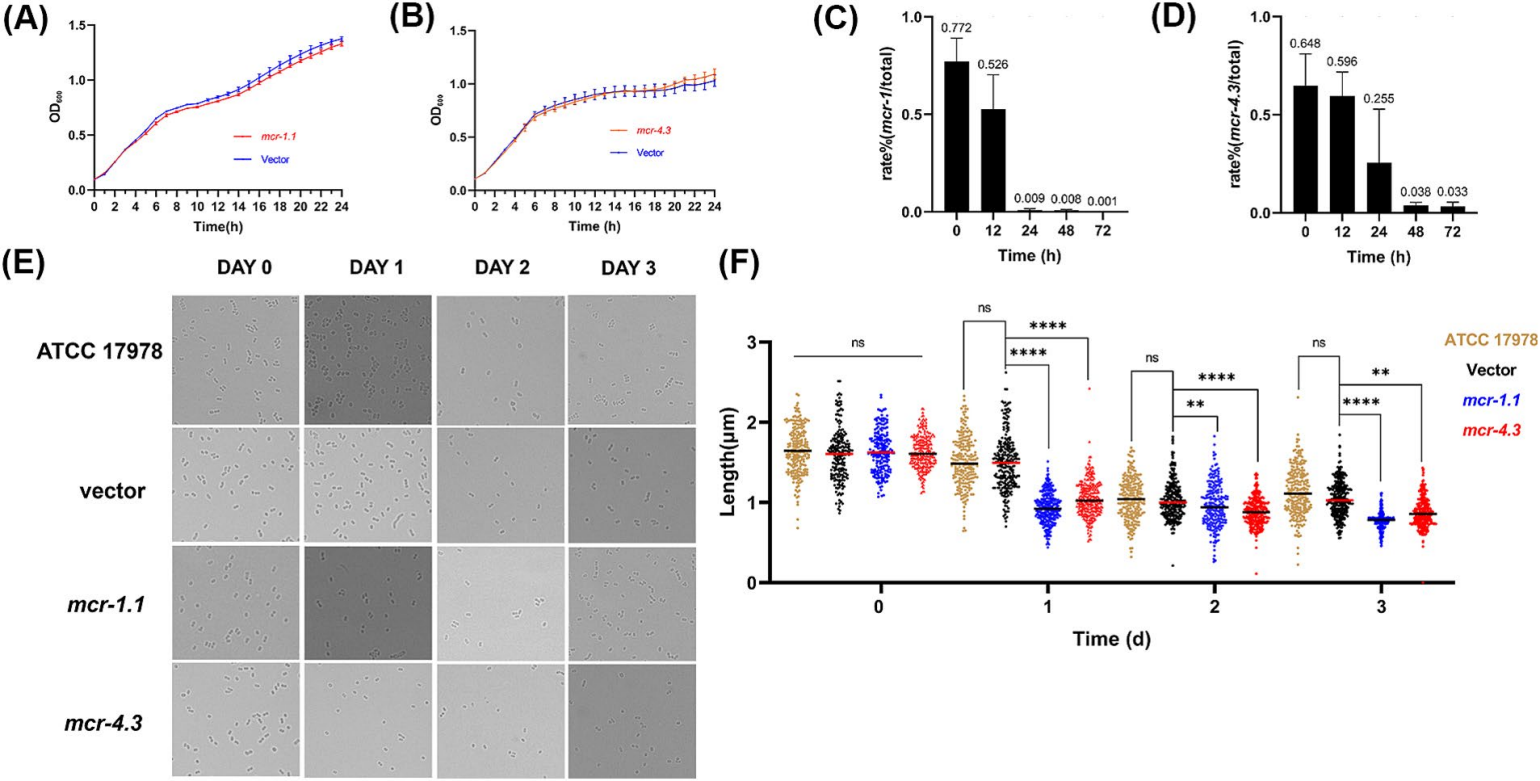
MCR affect the morphology and growth of *A. baumannii* in the stationary phase. **(A,B)** Growth curve of *A. baumannii* ATCC17978 carrying empty vector or expressing MCR were shown. **(A)** ATCC17978 carrying empty vector or expressing MCR-4.3; **(B)** ATCC17978 carrying empty vector or expressing MCR-1.1. The Y-axes showed optical densities at OD_600_ of broth cultures; X-axes showed time of growth (hours). **(C,D)** *In vitro* competition experiments were also performed to determine the effect of *mcr-1.1* and *mcr-4.3* expression on bacterial growth. Rate (%) = CFU _mcr_/ CFU _total_ * 100%. CFU total is all *A. baumannii* (culture on LB agar supplemented with gentamicin) and CFU mcr is from MCR-expressing *A. baumannii* (culture on LB agar supplemented with colistin/gentamicin). **(E)** Images of ATCC 17978 and ATCC 17978 carrying empty vector or *mcr* showing morphology variant were induced during the stationary phase where bacteria are subjected to increased survival pressures and the MCR-expressing *A. baumannii* was too weak to grow better (>200 cells for each group). **(F)** Scatterplots of cell length in ATCC 17978 (gold), Vector cells (black), MCR-1.1-expressing cells (blue) and MCR-4.3-expressing cells (red) at the indicated time points. Middle lines represented median values.

Bacterial cells were grown overnight in LB broth at 37°C. After the cell density was normalized (OD_600_ = 0.5), the resulting cultures were serially diluted, and 10 μL of each dilution was spotted onto LB agar or LB agar supplemented with the indicated concentrations of sodium dodecyl sulfate (SDS) and ethylenediaminetetraacetic acid (EDTA) (Figure 1A: 0.1% SDS + 0.2 mM EDTA or 0.1% SDS + 1.0 mM EDTA; Figure 1B: 0.1% SDS + 0.4 mM EDTA or 0.5% SDS + 0.4 mM EDTA). The plates were incubated at 37°C and photographed after ∼24 h (Feng et al., 2022). Experiments were performed with 3 biological replicates.

### 2.7 Whole-genome sequencing and SNP analysis

The genomic DNA of the F7-AB was extracted using the Qiagen Blood and Tissue kit (Qiagen, Hilden, Germany). DNA libraries were constructed with 350 bp paired-end fragments and sequenced using an Illumina HiSeq 2000 platform. The Illumina reads of the strain were mapped against the reference genome sequence of *A. baumannii* ATCC 19606 (GenBank accession: GCF_009035845.1) using the program MAQ. In our WGS analysis, we used a variety of programs for different steps. We started with FastQC (Babraham Bioinformatics, 2010) and Trimmomatic (Bolger et al., 2014) for quality control and preprocessing of raw sequencing data. Trimmed reads with length > 30 and Phred scores > 20 were retained for subsequent analyzes. Then, we employed BWA (Li and Durbin, 2009) for aligning the reads to a reference genome (*A. baumannii* ATCC 19606; GenBank accession: GCF_009035845.1). Variant calling was done using Genome Analysis Toolkit (GATK) (McKenna et al., 2010; DePristo et al., 2011). After obtaining the variant calls from the WGS data, we further filtered and annotated the SNPs using VCFtools (Danecek et al., 2011) and SnpEff (Cingolani et al., 2014) for SNP analysis. We excluded ambiguous calls as wells as indels and MNVs. We also excluded calls with low coverage using a minimum depth of either 10% of the mean coverage and calls with mapping quality or base quality scores < 30. Consistent with previous findings (Oikonomou et al., 2015; Nhu et al., 2016; Zhang et al., 2017; Nurtop et al., 2019; Jovcic et al., 2021; Kabic et al., 2023), four mutations [LpxD (E117K), PmrA (I13N), PmrB (A138T and A444V)] linked to colistin resistance were detected among the screened SNPs (Supplementary Table S6).

### 2.8 Colistin combination susceptibility testing

Synergy measurement was utilized to determine the antimicrobial effect of colistin and macromolecular hydrophobic antibiotics (rifampicin, erythromycin and cefoxitin) upon colistin-resistance *A. baumannii* (MCR-1.1-expressing cells, MCR-4.3-expressing cells, pig feces-derived MCR-4.3-expressing *A. baumannii* strain AB18PR065, clinical *A. baumannii* strain F7-AB). The checkerboard assay was set up in a 96-well plate. Briefly, columns 1 to 8 contained 2-fold serial dilutions of colistin, and rows A to H contained 2-fold serial dilutions of macromolecular hydrophobic antibiotics. Then the strains were cultured to exponential phase and adjusted to a density of OD_600_ = 0.5 by diluting with LB broth. After dilution at a ratio of 1: 10, 20 μL of diluted culture was added to 180 μL of LB broth containing colistin and another indicated antibiotics in a 96-well plate. A nanophotometer (NP80, IMPLEN) was utilized to measure the optical density at 600 nm (OD_600_) in each well before and after incubation at 37°C for 16 h. The fractional inhibitory concentration index (FICI) was calculated according to a previously published protocol (Pál et al., 2023). Briefly, FICI = (MIC of drug A in combination) / (MIC of drug A alone) + (MIC of drug B in combination) / (MIC of drug B alone), Synergy is defined as FICI ≤ 0.5, Indifference is defined as 0.5 < FICI ≤ 4, Antagonism is defined as FICI > 4 (Jenkins and Schuetz, 2012).

### 2.9 Synthesis of cDNA and quantitative real-time PCR

Exponentially growing bacterial cultures (optical density at OD_600_ = 0.4–0.6) of ATCC 17978 (*mcr-1.1*/pMMB67EH), ATCC 17978 (*mcr-4.3*/pMMB67EH) and ATCC 17978 (pMMB67EH only, as a control group) were pelleted. After the supernatant was removed, the cell pellet was resuspended in RNA-easy Isolation Reagent (Vazyme). The total RNA was precipitated by the addition of isopropanol and collected by centrifugation. The supernatant was discarded, and the RNA pellet was washed with 75% ethanol. After the pellet was air-dried, the mRNA was dissolved in RNase-free H_2_O. Any contaminating genomic DNA was digested with gDNA wiper Mix (Vazyme). The purified mRNA was reverse transcribed to cDNA with HiScript II qRT SuperMix (Vazyme). The cDNA levels of the target genes were then quantified with quantitative real-time PCR (qPCR) on CFX Opus 96 Real-Time PCR Instrument (Bio-Rad) using AceQ Universal SYBR qPCR Master Mix (Vazyme). All qPCR primers were determined to be >95% efficient, and the cDNA molecular masses were experimentally confirmed to be within the linear dynamic range of the assay. The signals were normalized to those of the housekeeping 16S rRNA transcript and quantified with the ΔΔCt method. The error bars are the 95% confidence intervals of three technical replicates.

### 2.10 Bright-field microscopy for measuring cell length

The *A. baumannii* strains were grown to mid-log phase (OD_600_ = 0.6–0.8) in LB medium. The cultures were pelleted, washed once with PBS, adjusted to an OD_600_ of 0.5 with PBS, and diluted 1: 10. Then, 200 μL of the diluted culture was added into 1800ul of LB. Cultures were incubated at 37°C with shaking (220 rpm). After 0, 1, 2, 3 day, bright-field imaging was performed on an inverted microscope (Olympus BX63). The ImageJ software was used to analyze the cell length.

### 2.11 Statistical analysis

Statistical analysis was performed using Prism (version 8.3.0, GraphPad Software). Data were analyzed using the paired Student’s *t* test, and for comparison of data from three or more conditions, analysis of variance (ANOVA) was used. *p* value of 0.05 or less was considered statistically significant.

## 3 Results

### 3.1 MCR affects the morphology and growth of *Acinetobacter baumannii* in the stationary phase

**Figure 2:**
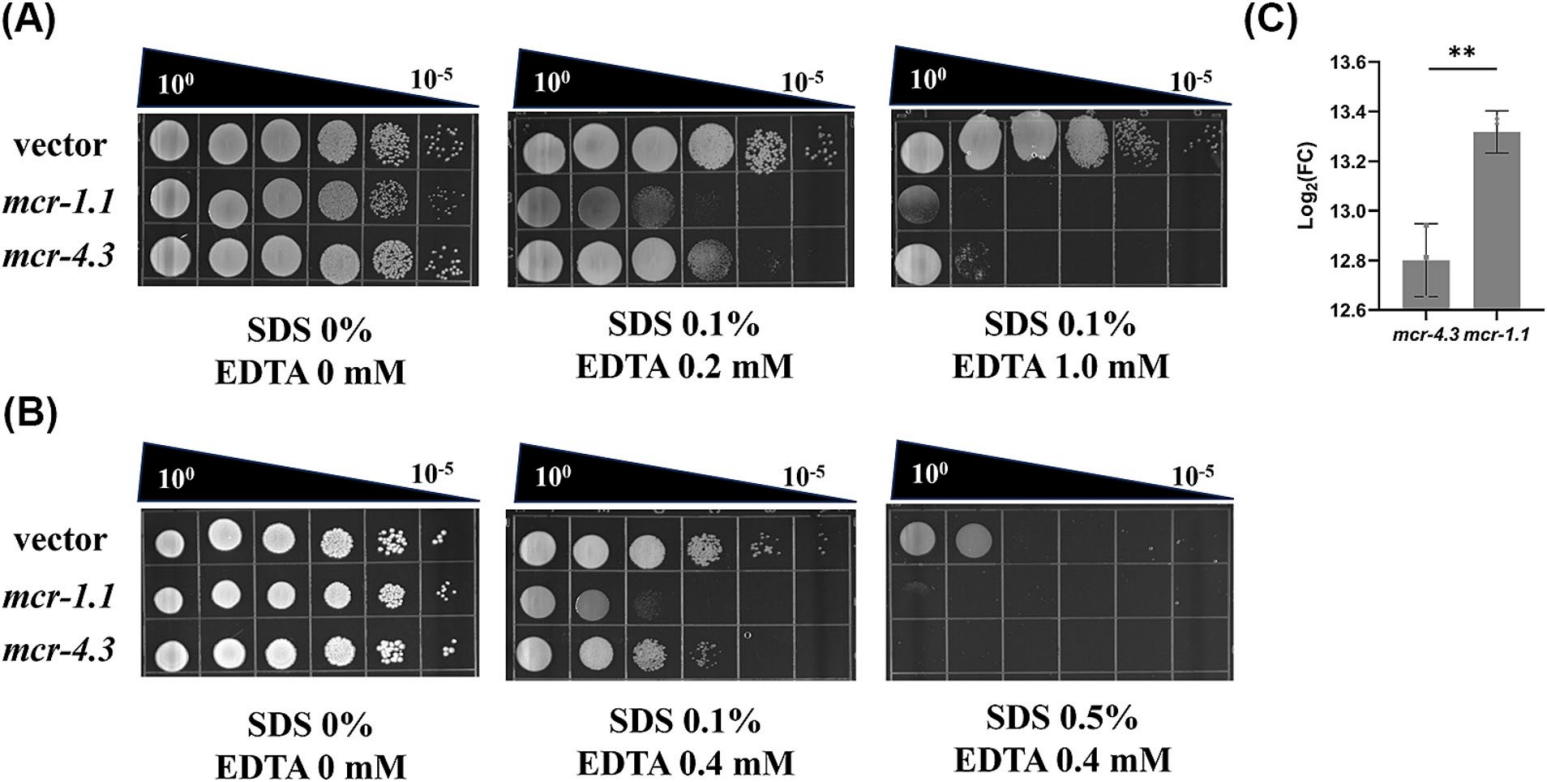
MCR confer an outer membrane permeability defect to *A. baumannii*. **(A)** Efficiency of plating assays on LB agar plates containing 0.1% SDS and 0.2 mM or 1.0 mM EDTA were shown on 10-fold dilutions of cultures. Cells expressing *mcr-1.1* and *mcr-4.3* were more sensitive to SDS/EDTA, indicating OM permeability impair. **(B)** Efficiency of plating assays on LB agar plates containing 0.1% or 0.5% SDS and 0.4 mM EDTA were shown on 10-fold dilutions of cultures. Each assay was repeated 3 times with similar results. **(C)** Levels of *mcr* mRNA in MCR-1.1-expressing or MCR-4.3-expressing *A. baumannii* ATCC 17978.

Although evidence of our previous study showed that expression of MCR-1 induces cell shrinkage and death in *E. coli* and *K. pneumonia* during the stationary phase (Feng et al., 2022), the impact of MCR on the viability of *A. baumannii* remains unclear. To study the impact of MCR on the physiology of *A. baumannii*, the plasmids pMMB67EH-NP-*mcr-1.1* and pMMB67EH-NP-*mcr-4.3* carrying *mcr-1.1* and *mcr-4.3* and their native promoters, respectively, were generated, and the low-copy number pMMB67EH-spy plasmid served as the empty vector control. We then constructed three strains, *A. baumannii* strain ATCC 17978 carrying pMMB67EH-NP-*mcr-1.1* (MCR-1.1-expressing cells), strain ATCC 17978 carrying pMMB67EH-NP-*mcr-4.3* (MCR-4.3-expressing cells) and ATCC 17978 carrying the empty vector pMMB67EH-spy (Vector cells). Minimum inhibitory concentration (MIC) of colistin against the three strains above were then determined (Table 1), and the growth of MCR-1.1-expressing cells, MCR-4.3-expressing cells and Vector cells in LB medium were monitored (Figures 2A,B). Like previously observed in the *E. coli* (Feng et al., 2022), the expression of MCR did not affect the growth of *A. baumannii* ATCC 17978 (Figures 2A,B). However, when we extended the incubation time, we found that the MCR-expressing cells have shorter length compared to vector cells in the stationary phase (Figure 2E). Meanwhile, *in vitro* competition experiments were also performed to determine the effect of *mcr-1.1* expression on bacterial growth in the stationary phase. As shown in Figures 2C,D, after 24 h, the growth rates of *A. baumannii* strains expressing MCR showed significant decrease when compared to Vector cells (pMMB67EH-spy, without *mcr*). These results suggest that the viability and competitiveness of *mcr*-positive *A. baumannii* strains was impaired in the stationary phase.

**Table 1:** Minimum inhibitory concentrations of antibiotics tested.

MIC of (μg/mL)	ATCC17978	ATCC17978 (vector)	ATCC17978 (*mcr-1.1*)	ATCC17978 (*mcr-4.3*)	AB18PR065	F7-AB
Colistin	2	2	8	8	8	>256

### 3.2 MCR impairs OM permeability in *Acinetobacter baumannii*

**Figure 3:**
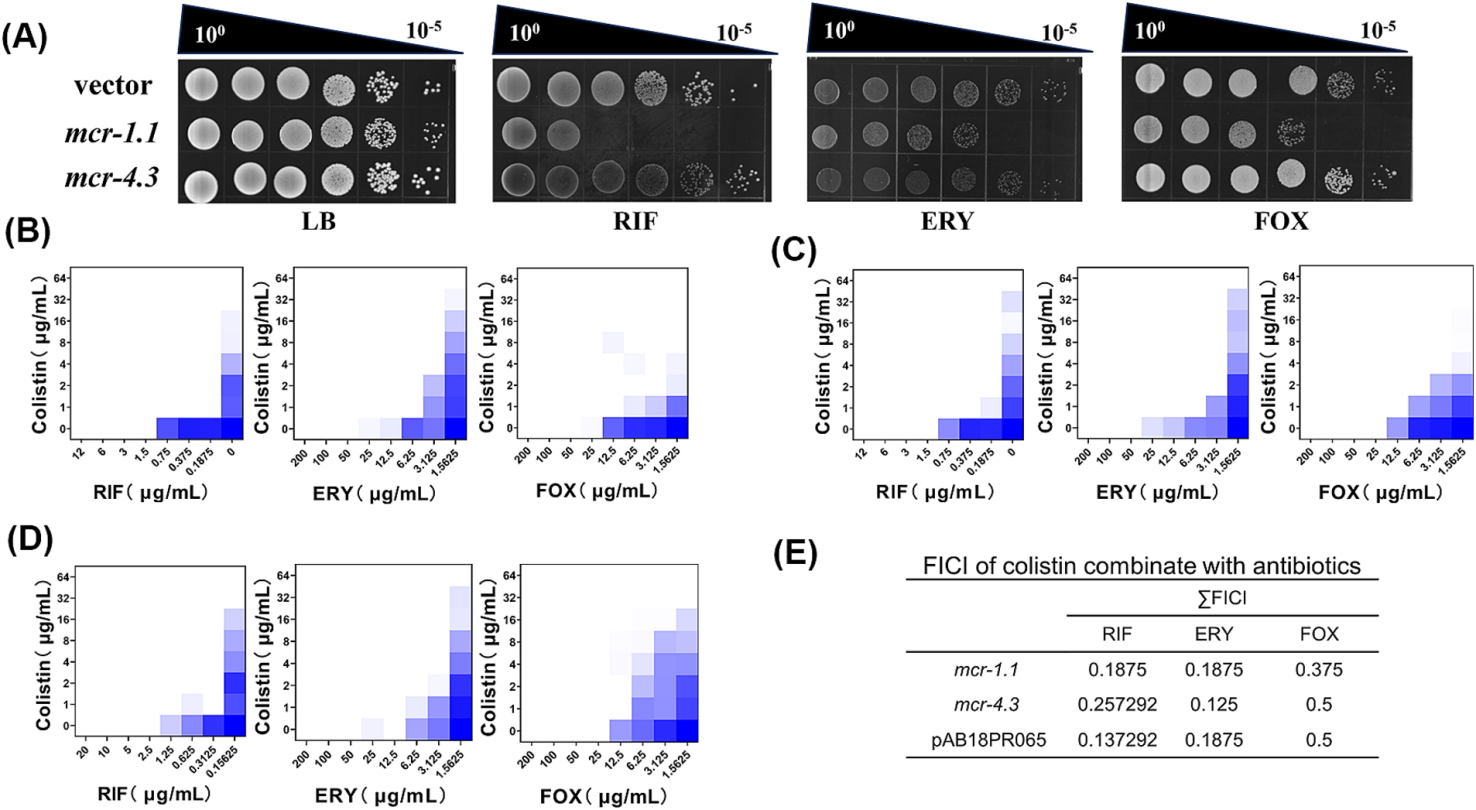
Colistin potentiates macromolecular hydrophobic antibiotics in *mcr*-positive *A. baumannii.* **(A)** Efficiency of plating assays on LB agar plates with 0.75 μg/mL RIF, 10 μg/mL ERY, 15 μg/mL FOX. Ten-fold dilutions of cultures are indicated above the left plate. MCR-expressing cells exhibited increased sensitivity to macromolecular antibiotics. **(B–D)** Checkerboard broth microdilution assays showing dose-dependent potentiation of rifampicin, erythromycin and cefoxitin by colistin against *A. baumannii* expressing **(B)** MCR-1.1; **(C)** MCR-4.3; and **(D)** a pig feces-derived MCR-4.3-expressing *A. baumannii* strain (AB18PR065). The calculated values of FICI are listed in **(E)**. **(E)** The FICI is the sum of the two FICIs, with an FICI index ≤0.5 considered synergistic. Dark blue regions represent higher cell density. Data in **(B–D)** represent the mean OD_600_ of two biological replicates. RIF, rifampicin; ERY, erythromycin; FOX, cefoxitin.

To understand how MCR damage the viability of *A. baumannii* strains, we investigated the mechanism underlying this phenomenon. Overexpression of *mcr-1* results in significant degradation in cell membrane and cytoplasmic structures (Yang et al., 2017). Therefore, we wonder whether the bacterial death caused by MCR in *A. baumannii* strains results from increased OM permeability. To test this, we employed SDS/EDTA sensitivity assays to measure the OM permeability of MCR-1.1-expressing cells, MCR-4.3-expressing cells, vector cells (Figures 3A,B). As expected, all the MCR-expressing cells displayed increased sensitivity to detergents SDS/EDTA compared with the vector cells, indicating that MCR-1.1 and MCR-4.3 both increased the permeability of OM in *A. baumannii*. Moreover, MCR-1.1-expressing cells were more sensitive to SDS/EDTA than MCR-4.3-expressing cells, which may be associated with the different expression level of MCR protein (Figure 3C). These data demonstrated that the increased OM permeability affected the viability of *mcr*-positive *A. baumannii* strains in the stationary phase.

### 3.3 Colistin potentiates antibiotics in *mcr*-positive *Acinetobacter baumannii*

*Acinetobacter* is intrinsically resistant toward many antibiotics because of the low permeability of its outer membrane (Zahn et al., 2016), especially resistant to OM-impermeable large molecule antimicrobials due to the absence of large-channel general porins such as OmpF and OmpC (Sugawara and Nikaido, 2012). Therefore, we speculated that *mcr*-positive *A. baumannii* might be more sensitive to certain antimicrobials. To do it, we screened nearly 10 antibiotics (Supplementary Table S3), covering most of macromolecular hydrophobic antibiotics for change in MIC with or without the presence of MCR protein. As we had expected, we found that MCR-expressing cells were more sensitive to rifampicin (RIF), erythromycin (ERY), and MCR-1.1-expressing cells were more sensitive to cefoxitin (FOX), in contrast to MCR-negative cells (Figure 1A). Next, we aimed to investigate whether antibiotics could potentiate this effect. In the presence of colistin, several antibiotics were highly potentiated. Checkerboard broth microdilution assays showed potentiation of RIF, ERY, and FOX by colistin against MCR-expressing *A. baumannii* (Figures 1B–D). By calculating their Fractional Inhibitory Concentration Index (FICI), in which ≤0.5 indicating synergistic, we found that there was potent synergy of colistin combination with rifampicin, erythromycin and cefoxitin versus MCR-expressing *A. baumannii* (Figures 1B–D).

To investigate whether colistin potentiation is conserved beyond laboratory-generated MCR-expressing *A. baumannii*, we tested the FICI of a MCR-4.3-expressing *A. baumannii* strain (AB18PR065) derived from pig feces (Ma et al., 2019) in colistin combination with rifampicin, erythromycin and cefoxitin. As expected, the FICI was less than or equal to 0.5 for all three drugs (Figures 1D,E), indicating that MCR-4.3 also rendered the potentiation of large molecule antimicrobials in clinical *A. baumannii* expressing MCR.

### 3.4 Colistin potentiates RIF in clinical colistin-resistance *Acinetobacter baumannii*

**Figure 4:**
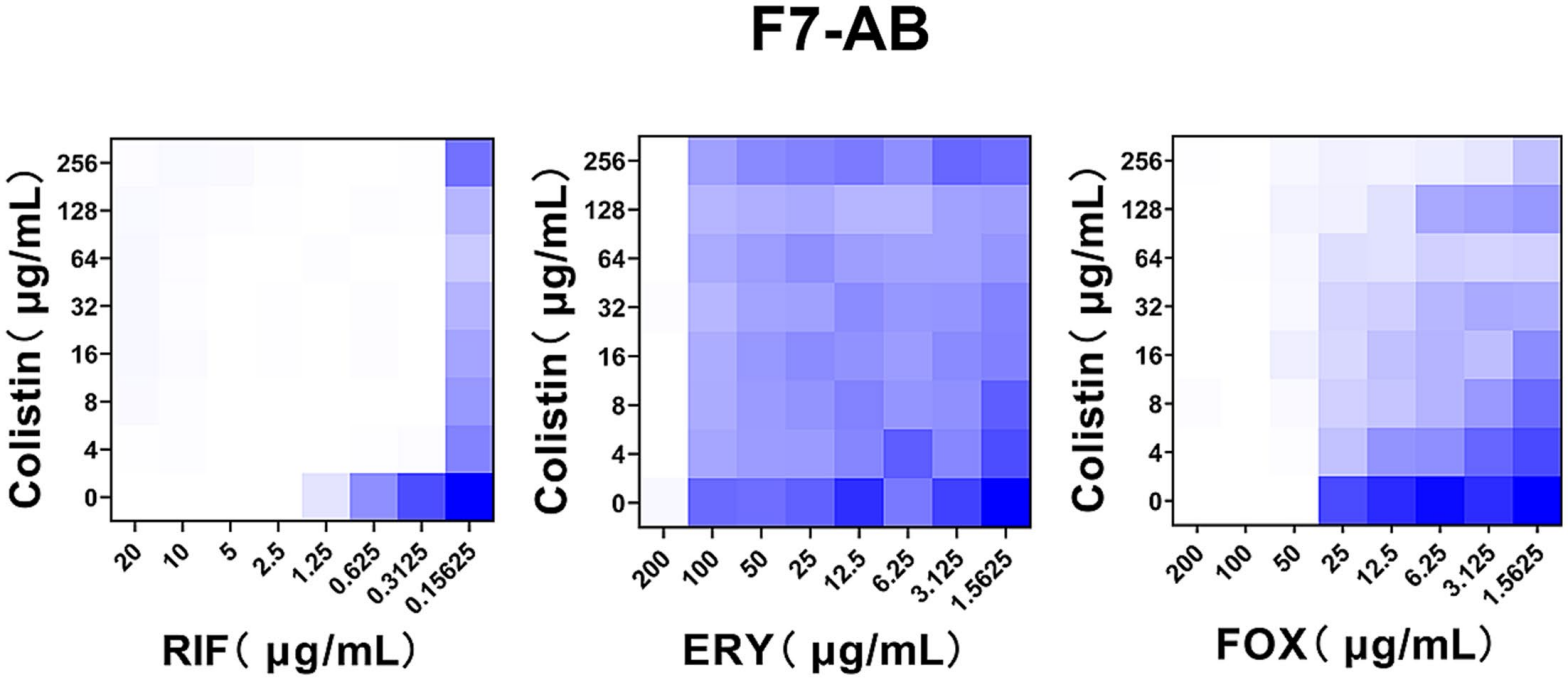
Colistin potentiates RIF in clinical colistin-resistance *A. baumannii.* Checkerboard broth microdilution assays showed dose-dependent potentiation of rifampicin but not erythromycin and cefoxitin by colistin against clinical *A. baumannii* strain F7-AB which is intrinsically resistant to colistin. Dark blue regions represent higher cell density. Data represent the mean OD_600_ of two biological replicates. RIF, rifampicin; ERY, erythromycin; FOX, cefoxitin.

Given the therapeutic potential of combination therapy in *mcr*-positive *A. baumannii,* we wondered whether this potentiation could be applicated to clinical chromosomally mediated colistin resistance *A. baumannii*, since the complete loss or modifications of the target LPS are two main mechanisms of chromosomally mediated colistin resistance *A. baumannii* (Novović and Jovčić, 2023), which is similar to the acquired mechanism of *mcr* encoding a phosphoethanolamine (pEtN) transferase that adds pEtN to the lipid A component of LPS. To test this, we fist the FICI of a clinical *A. baumannii* strain F7-AB which is intrinsically resisted to colistin [LpxD (E117K), PmrA (I13N), PmrB (A138T and A444V)] (Oikonomou et al., 2015; Nhu et al., 2016; Zhang et al., 2017; Nurtop et al., 2019; Jovcic et al., 2021; Kabic et al., 2023). Encouragingly, the result of the checkerboard assay was similar with that of mcr-positive *A. baumannii* except erythromycin and cefoxitin (Figure 4). These observations indicate that the combination of colistin with rifampicin is not only able to overcome *mcr* mediated acquired colistin resistance, but also able to treat chromosomal colistin resistance.

## 4 Discussion

In this study, we were aimed at better understanding impact of MCR on *mcr*-positive *A. baumannii* in the stationary phase and bridging the gap between widespread colistin resistance and the development of new treatments. Our data founded that MCR shortened the cell lengths and decreased the competitiveness of *A. baumannii*. Then using SDS sensitivity assay, we found that MCR provides serious disruption to the OM of *A. baumannii*, which was thought to be resistant to many large molecule antibiotics due to its lower membrane permeability (Zahn et al., 2016). Therefore, we used antimicrobial susceptibility testing (AST) and checkerboard assay to find that although *mcr-1.1/mcr-4.3* confers no change in susceptibility to all selected antibiotics, colistin and rifampicin, erythromycin and cefoxitin combination therapy shows efficacy against colistin-resistance *A. baumannii*.

A recent study reported that MCR-1 damages the OM permeability barrier by disrupting lipid homeostasis in *E. coli* and *K. pneumonia*, resulting in cell shrinkage and death during stationary phase (Feng et al., 2022). Furthermore, overexpression of MCR results in impaired competitive ability and drastic degradation in Gram-negative cell membrane (Yang et al., 2017). The same phenomena were observed in our study on the outer membrane of *mcr*-positive *A. baumannii.* Given the significance of *A. baumannii* infection and its prevalence in hospital setting (Magill et al., 2014; Lob et al., 2016), we wonder whether the strains expressing MCR will be more vulnerable than vector cells so that the colistin-resistance cells could be eliminated by some large antibiotics capable of penetrating the cell membrane. Unexpectedly, the susceptibility of intracellular antibiotics does not change when cells expressed MCR-1.1 or MCR-4.3. This observation is contrary to previous reports that the large OM-impermeable antimicrobials such as vancomycin, rifampicin and erythromycin would be more lethal to gram-negative bacteria with severe damaged outer membrane (Muheim et al., 2017; Mecsas et al., 2021). Indeed, more and more evidences support that OM permeability impaired strains need more stress conditions to display decreased growth rate, cell viability, competitive ability and more sensitive to large-scaffold antibiotics (Heesterbeek et al., 2019; Mecsas et al., 2021; Feng et al., 2022). And the stressful environment can be osmotic stresses (Ohnishi et al., 2004; Virolle et al., 2018), oxidative stress responses (Sun et al., 2014; Mancini and Imlay, 2015) and starvation in stationary phase. Accordingly, we show that MCR did not damage the growth of *A. baumannii* during the first day of culture, but impair the cells viability in the stationary phase.

Colistin acts by associating with the anionic LPS component of the Gram-negative outer membrane, causing membrane destabilization that leads to cell envelope permeability, leakage of cellular contents, and ultimately lytic cell death (Schindler and Osborn, 1979). When in combination with colistin, RIF or ERY can overcome *mcr-1* mediated colistin resistance in *Enterobacteriaceae*. Likewise, we found that as Gram-negative bacteria, *mcr*-positive *A. baumannii* is more sensitive to the combination of colistin and RIF or ERY. In this case, we guess that colistin just like a stress which aggravate the OM damage in *mcr*-positive *A. baumannii,* then large antibiotics like RIF can enter the cells and function more efficiently. Similar to the mechanism of resistance mediated by *mcr,* colistin resistance cause by chromosomal mutations include complete loss of LPS by inactivation of the biosynthetic pathway or modifications of target LPS driven by the addition of pEtN moieties to lipid A (Adams et al., 2009; Moffatt et al., 2010; Olaitan et al., 2014). The protective function of outer membrane that serves as a barrier is dependent on LPS and the OM permeability will increase when LPS was changed (Sherman et al., 2018). On this account, our data suggested that the combination of colistin with rifampicin also have the ability to overcome chromosomal colistin resistance in clinical *A. baumannii* strains F7-AB.

In conclusion, our findings suggest that *mcr-1.1* and *mcr-4.3* can induce OM defect in *A. baumannii* then leading to growth inhibition. Due to the increased permeability, synergy with colistin potentiates rifampicin in colistin-resistance *A. baumannii.* These findings would help develop strategies to improve the therapeutic effects of colistin-resistance *A. baumannii* infection.

## Data Availability

The datasets presented in this study can be found in online repositories. The names of the repository/repositories and accession number(s) can be found at: https://www.ncbi.nlm.nih.gov/, PRJNA1138508.

## Supplementary material

Figure S1 The map of plasmids and recombinant plasmids used for construct *mcr*-positive *A. baumannii* strains. **(A)** pMMB67EH-spy; **(B)** pMMB67EH-NP-*mcr-1.1*; and **(C)** pMMB67EH-NP-*mcr-4.3.*
