# Supplementary figures and images for: Outer membrane permeability of mcr-positive bacteria reveals potent synergy of colistin and macromolecular antibiotics against colistin-resistant Acinetobacter baumannii
Source: Front Microbiol. 2024 Nov 19;15:1468682. doi: 10.3389/fmicb.2024.1468682 (PMC11611826; doi:10.3389/fmicb.2024.1468682)

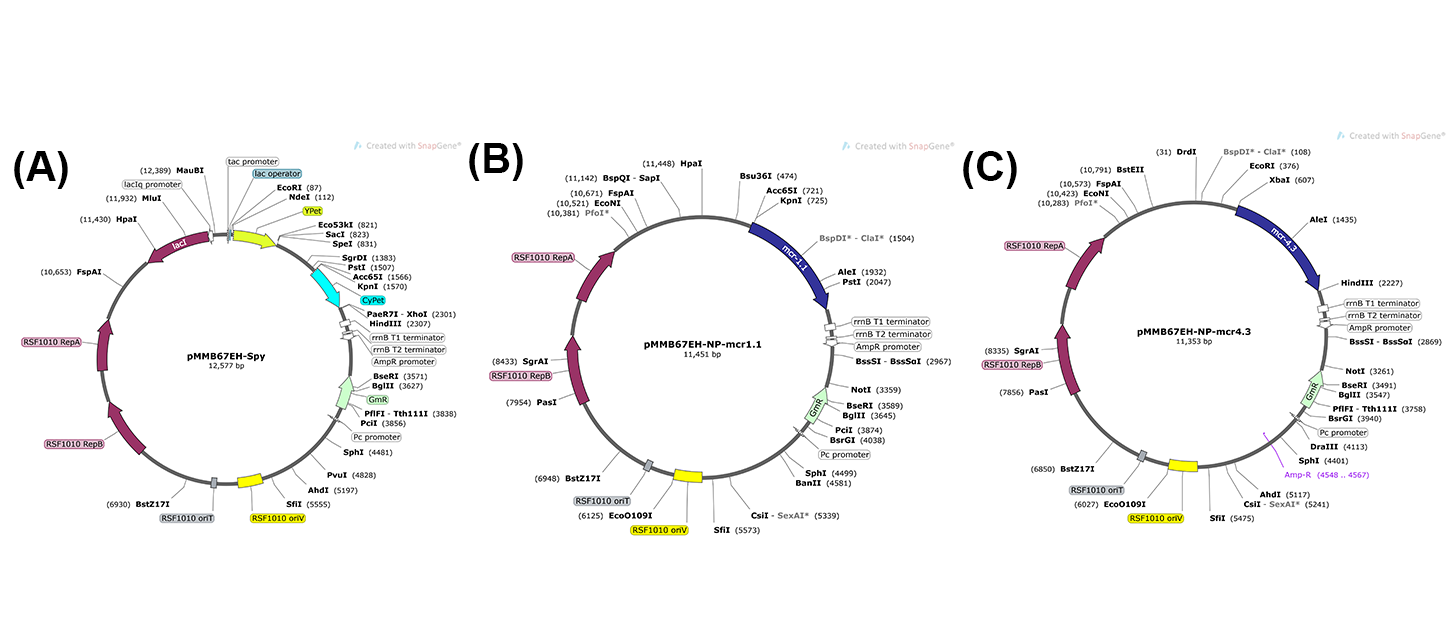

Supplement: Figure S1 — The map of plasmids and recombinant plasmids used for construct mcr-positive A. baumannii strains. (A) pMMB67EH-spy; (B) pMMB67EH-NP-mcr-1.1; and (C) pMMB67EH-NP-mcr-4.3. [file Image_1.tif]
